# *“I have to resist simply to exist”*: Black Physician Trainees’ Experiences of Professional Resistance

**DOI:** 10.5334/pme.1788

**Published:** 2025-04-29

**Authors:** Isaiah Horton, Kirsten Brown, Ting-lan Ma, Tasha R. Wyatt

**Affiliations:** 1 Department of Medicine, Uniformed Services University, Bethesda, MD, USA; 2 Department of Health Professions Education, Uniformed Services University, Bethesda, MD, USA

## Abstract

**Introduction:**

In medical education, acts of professional resistance have been studied across all racial and ethnic groups as an antidote to the social harm and injustice festering in medical education. However, not everyone experiences medical education in the same way; some groups experience it quite differently because of their social positions. In particular, Black physicians face anti-Black racism in medical education, which has the potential to shape their resistance in a particular way. This study was designed to understand what professional resistance looks like in Black residents/fellows in North America and how being Black shaped their experiences of resistance.

**Methods:**

This qualitative study used Endarkened storywork to understand how Black GME physicians experienced acts of resistance. Endarkened storywork is a Black-centered approach to research and a way of reclaiming authority to create knowledge. It weaves Endarkened feminism, Afrofuturism, and Indigenous storywork to center storytelling as essential to Black ways of being. We conducted 14 semi-structured interviews and analyzed the data using thematic, theoretical, and emergent coding through the constructs of Re-storying, Endarkened Storywork, and Black quilting.

**Results:**

Black trainees’ stories of resistance produced three quilting blocks to illuminate their experiences. First, resistance means continuing to exist in medicine despite all of the profession’s efforts to eradicate Black physicians. Second, they work tirelessly to improve Black health and facilitate opportunities for the future of Black people to have a career in medicine. Third, their resistance must contend with a long history of anti-Black racism, which takes a significant toll on the emotional and mental well-being of Black trainees in ways that are best described as racial battle fatigue.

**Discussion:**

For Black trainees, professional resistance is nuanced and calls into the present a long history of anti-Black racism. Their resistance includes occupying space in medicine, securing a future where Black people exist as physicians, and resisting the emotional burden of doing this work. While all forms of professional resistance are worthy of study, researchers should pay particular attention to how it manifests in various racial groups to understand the nuances of different strategies.

Institutions and individuals across North America harbor a deep commitment to anti-Black racism, and the proliferation of white supremacy [1,2,3,4,5,6,7,8,9]. The presence of these two ideologies in our social structures and systems is frequently attributed to North America’s historical relationship and entanglement with the transatlantic slave trade and European colonialism [10]. White supremacy, and its corollary of anti-Black racism is particularly tenacious in North America because it works within economic and political constructs to protect the rights and artifacts of people with the ‘right’ skin color, educational background, and pedigree, thus disadvantaging all other groups [9]. In higher education, white supremacy is found in curriculum choices, faculty-student interactions, and leadership decisions [2,3,5,11].

In medical education, where white supremacy is particularly strong [7,8], there are many historical incidences of the mistreatment of Black individuals, including the use of Black bodies to advance medical knowledge. Examples include the Tuskegee Syphilis study, Henrietta Lack’s cancer cells being taken without her consent to advance biomedical research, and the father of gynecology, J. Marion Sims, conducting repeated experimental surgeries on enslaved women without anesthesia. This, coupled with nearly a century of medical schools limiting the profession to wealthy, white, cisgendered males [12,13], has resulted in disproportionately low rates of practicing Black physicians. Currently, 13% of the U.S. population is Black; yet, only 5% of physicians are Black, and since 1978 the total number of Black male physicians has decreased [14]. Black physicians experience hypervisibility and pressure to represent the entire racial group, while also remaining simultaneously hyper-visible as Black bodies and invisible as physicians [13,15,16,17]. Further, understanding anti-Black racism is imperative to medical education because strands of oppression are interconnected and social determinates of health are associated with unequal access to resources that affect patients and providers.

However, in spite of the ongoing messages of anti-Black racism in medical education, there is a robust body of literature indicating that Black medical professionals, medical students, physicians in training, and staff physicians, resist much of the anti-Black racism they encounter [18,19,20,21,22,23,24,25]. Researchers assert that Black resistance has its own nuance and texture and is a unique, “radical tradition” grounded in both individual and collective responses to slavery, colonization, capital, and racial domination experienced throughout history and across the diaspora. That is, Black people “brought the past” with them, in the form of “cultures, critical mixes and admixtures of language and thought, of cosmology and metaphysics, habits, beliefs, and morality”, all of which were able to withstand society’s attempts to exploit and eradicate Black cultural substrates and populations [10, p. xiv].

Given this history, we suspected that there may be a nuanced and textured understanding of professional resistance among Black physicians who encounter social harm and injustice. Ellaway and Wyatt [26] define acts of professional resistance as: “*Individual and collective expressions of condemnation of social harms and injustices, with the intent of stopping them, preventing them from recurring, and/or holding those responsible to account*.” However, this definition does not account for the fact that there may be differences among various racial and ethnic groups for how they resist social harm and injustice in medical training and practice. As noted earlier, the Black community “brought the past” with them and may be influencing how Black physicians work to create change.

In this study, we explore the experiences of Black trainees as they engage in professional resistance against social harm and injustice. We focus on their efforts to reclaim and honor the past, challenge negative narratives in the present, and envision new possibilities in the future [27]. Further, by understanding their particular means of engaging in resistance, we hope that other medical education researchers will capture acts of professional resistance that are grounded in the specific sociopolitical contexts of other groups. Guiding our study were the following research questions: *How do Black Graduate Medical Education (GME) physicians experience the phenomenon of professional resistance? What are the personal and professional effects of professional resistance for Black trainee physicians?*

## Methods

In this study, we used endarkened storywork to understand how Black GME physicians experienced acts of resistance [27]. Endarkened storywork is a Black-centered approach to research; it is a way of reclaiming authority to create knowledge. This approach weaves Endarkened feminism, Afrofuturism, and Indigenous storywork to center storytelling as essential to Black ways of being [27]. We chose Endarkened storywork because it aligns with key tenets of Black resistance including community, culture, and chronology. Whereas most research is based on methodological frameworks created by, and centered within whiteness, even critical theory, Endarkened storywork centers Black ways of knowing. For example, rather than assuming a researcher/object relationship which is inherent in positivism, or engaging in reflexivity which is standard in constructivism, Endarkened storywork assumes connectivity and responsibility between scholars and the communities that they produce knowledge with. Endarkened storywork allows for the exploration of linguistic and “trickster” technologies—that is the use of linguistic codes (e.g., code switching) and hacks (e.g., malicious compliance). Further, although white ways of knowing assume a time-bounded or current framing of knowledge, Endarkened storywork highlights the importance of ancestors and future generations, to connect “collective experiences across time and space” [27, p. xxvi].

Further, as Toliver [27] described, Endarkened storywork “is a method of nurturing, not countering” (p. xix). This methodology enables researchers to explore accomplishment, affirmation, and community, rather than solely addressing anti-Blackness or racism. That is, Endarkened storywork does not require research questions, data analysis, or findings be positioned in relation to, or in conversation with, whiteness. For example, research questions about Black trainees’ experiences of resistance, written from a critical framework would likely include aspects of power, racism, or anti-Blackness. However, by using an Endarkened storywork approach these concepts are still considered if the researcher wants but does not require a focus or conversation with those topics. Rather, the research questions and data analysis can focus on community, connectivity, or allegory as criteria that stand alone.

By using a methodology that does not center white power structures inherent in qualitative research (e.g., finding themes across participants stories), we answer the call for transdisciplinary, critical race research within health professions education (HPE) that is epistemologically and methodologically consistent [7]. This approach also illustrates how researchers can conduct qualitative analysis without relying on traditional methods like grounded theory or thematic analysis. We acknowledge that some readers may feel discomfort with our methodological choices; however, such discomfort is often a response when whiteness is no longer the standard. We encourage readers to sit with this discomfort and learn from it.

### Participants and interviews

Participants were recruited through messages posted in Black GME groups, social networks, snowball sampling, and personal invitations. Together, these approaches yielded 14 participants from 10 institutions. The research team decided that the first author (a Black male and the only Black person on the research team) would interview the participants because research shows that interviews are qualitatively different depending on the race of the interviewer and participant [28]. The semi-structured interview guide (see Appendix A) was developed based on existing literature [29], the first author’s (IH) personal experiences, and threads that we saw within the data. For example, questions about minority tax and racial battle fatigue were drawn from previous research, questions about survival and protecting one’s mental and physical health were informed by IH’s lived experiences, and questions about how bodies are perceived and categorized in medical spaces emerged from early coding of the transcripts [19,24,30,31,32].

The institution IRB permits waiving the formal consent form to enhance participant protection. As a result, we do not retain their names on paper records for this study on resistance. This approach was approved by the IRB, which deemed the study exempt. As part of the approved protocol, we obtained oral informed consent from all participants and reviewed a study information sheet before the interview, to uphold the principles of voluntary participation prior to data collection. IH conducted all racially concordant interviews via an online platform between October 2023 and July 2024. With participants’ permission interviews were recorded. Initial transcripts were created using the automated transcript function and were subsequently cleaned by IH and KB. We began to notice indications of data sufficiency after the 12th participant and continued until participant 14. To maintain confidentiality, all demographic information is reported in aggregate form (see Table 1).

**Table 1:** Sample Demographics.


CHARACTERISTIC	VALUE: NUMBER (%)

**PYG Year**	

PYG 1	0 (0)

PYG 2	4 (28.6)

PYG 3	2 (14.3)

PYG 4	2 (14.3)

PYG 5	4 (28.6)

PYG 6	2 (14.3)

**Specialty**	

Surgical	5 (35.7)

Non-Surgical	9 (64.3)

**Training Level**	

Resident	9 (64.3)

Fellow	5 (35.7)

**Gender**	

Woman	4 (28.6)

Man	10 (71.4)

**Military**	

No	8 (57.5)

Yes	6 (42.9)

**Sexual Orientation**	

Heterosexual	7 (50.0)

Gay	3 (21.4)

Unknown/did not disclose	5 (35.7)

**Religious Affiliation**	

No	8 (57.1)

Yes	6 (42.9)


### Data analysis

We started data analysis by using what we knew, traditional inductive and deductive academic coding methods [33]. Each team member independently coded the transcripts and wrote memos, with bi-weekly meetings to discuss the project. Initially our coding was iterative, allowing insights from earlier transcripts to inform later interview questions. During team meetings, white research team members (TW and KB) focused on being a listener while TM took notes. Toliver [27] used the metaphor of Black quilting to describe data analysis, but as a team we struggled with how to understand this metaphor. However, we recognize that Toliver explicitly chose not to use prescribed academic analysis techniques, such as open, focused, and selective coding—precisely the approach we had been using. Yet, no one on the research team quilts, so we needed to learn about Black quilt making.

We started by learning from the African American Quilters of Baltimore (AAQB) because historically Black quilt making was place-based and two of the authors live in this geographic area. We then expanded out, based on resources from the AAQB and Women of Colors Quilters Network (WCQN). IH and KB listened to lectures, personal stories, and tutorials from Black quilters; we explored videos, followed accounts on social media, and read oral history transcripts. Appendix B provides a sample of the resources that we used and a list of key ideas that we learned.

Black quilts are created via piecing and applique, can be used to communicate meaning, and do not hold to the expectation of uniformity or regulation [27]. To engage in analysis, Toliver suggested that researchers follow threads, find seams of convergence, and consider patches in both their individual piecing and larger picture. Key insights from Toliver’s text and our Black quilting research included the use of existing fabric, techniques for efficiency, the value of creativity and problem-solving, and the idea that function (i.e. warmth) is prioritized over perfection.

We faced challenges in managing the depth and richness of our data set; Toliver’s quilt was book length, and we found it difficult to cut our analysis down to a pocket sized square. To address this, we focused our analysis on topics that IH thought were most important. In quilting terms, these topics became our “blocks”, they are cut and used with intent. The original codes, which we now saw as “scraps” from our initial coding, included an early structure IH created to address the research questions and a sorting of codes by participant characteristics that KB used to identify patterns. IH combined these blocks and scraps to write an initial draft of the findings, which the team then reviewed and refined collaboratively. In a follow-up meeting, IH and KB discussed the analysis further; KB generated a draft of a table (see Appendix C) based on IH’s notes, and TM edited the table. We focused on the threads or points of connection. These threads became key parts of the findings that we present below.

Given the absence of a template or exemplars for Endarkened storywork in the HPE literature, we provide the table in Appendix C as a worked example. Black quilters simultaneously talked about traditional ideas of precision (e.g., stitch size, seam alignment) while valuing imperfect or creative methods that saved time (e.g., tying knots). Likewise, several participants in this study talked about “not slipping” or “needing to always be excellent,” and in the same space noted how the stress of perfection would shorten their lives, cause burnout, or was otherwise problematic because their white colleagues were allowed to make mistakes without it reflecting the whole race. Therefore, rather than cleaning up our analysis into neat, aligned columns, we choose to show the imperfections which is why there is not a congruent arrangement of participants’ quotes, scraps, pieces, and threads. This example is by no means ideal, nor do we claim fidelity with Toliver’s metaphor; we are not quilters. At best, this is our attempt at centering Blackness in data analysis and a step towards making the metaphor of Black quilting more tangible.

### Quality and reflexivity

At the time of this study, all authors were engaged in HPE research as either students or faculty. IH is a Black, cis, man internist. His goal is to center Black and other marginalized experiences in health professions research. He wishes for marginalized people to be seen in research and to give insight to allies and advocates on how to better support them. KB is a white, queer, cis, disabled social science researcher. TM is a social science researcher focusing on medical trainees’ mistreatment, burnout, and wellbeing. She is a first-generation immigrant and engages in multiple forms of resistance against geopolitical oppression. TW is a white, cis, woman who studies acts of professional resistance in HPE. Collectively our positionalities shaped this study via the methods we used and the analytic tools we brought. For example, we decided to have the first author collect interview data so that participants would feel greater rapport and comfort. We also decided to learn about Black quilting, rather than engage in the well-worn academic path of thematic analysis. The study protocol was approved by Uniformed Services University’s IRB and was conducted between August 2023 and May 2024.

## Results

### Existence As Resistance: *“This is a space that is historically reserved for a certain type of person”*

Existing within a white supremacist profession was the first block of the quilt. Participants did not see their resistance as something separate from them, rather they described it as all-encompassing of their experience as Black trainees in medicine: “My whole life is a resistance” (P3) and “I have to resist simply to exist” (P8). Given that there are so few physicians who look like them, just being Black in medicine creates its own kind of resistance, one that does not even need to rely on overt actions of challenge, as participant 14 explained, “The simple act of being Black in these spaces is my form of resistance right now.” Being a Black resident in medicine necessitated that participants constantly carve out spaces for themselves amidst the prevailing experience of:

People don’t think you deserve to be [here]… You don’t feel wanted and welcome [and] it is almost every day. It’s just torture. But you push through because it’s not just about you. It’s about setting an example for others who can see somebody that looks like you. (P12)

As they attempted to exist in their programs, participants described experiencing ongoing mistreatment especially when compared to their white colleagues. These experiences included being ignored by white staff and peers, disproportionately questioned and doubted by colleagues and superiors, and double standards where peers put forth attitudes of “you can’t question me, but I can question you” (P12). However, it did not stop there; communication modes were called into question; P9 was berated for texting his supervisor, even though his white colleagues were allowed to communicate in this manner. More severe experiences included participants who described public humiliation from superiors and attacks on their clinical and academic skills (P10, P11, P13). When participants raised issues with the differential treatment they received, one described the advice a Black superior gave them, which was essentially there was not much they could do.

‘When these things happened,’ he said ‘You have to be of a certain complexion to be taken seriously.’ That was kind of tough to swallow at first, but this is coming from someone who’s been doing this for over 40 years. (P10)

This block of the quilt also incorporates sections where residents discussed institutional pressure to uphold whiteness. For example, standards of white appearance were sewn into their stories, typically in how they wore their hair (P9, P10, P14) or moved their bodies (P6, P13). Participants described feeling the push to hide their Blackness; P13 explained how he needed to be a certain way to exist in medicine: “I feel like every day I go in and I’m hyper aware of my size, my height, the tone of my voice, how I greet people.” P13 attributed body consciousness to the fact that “White people oftentimes don’t understand Blackness and they have responses based in fear due to that.” As a result of white fear, participants exerted extra energy into identity management.

I could see it in their face…[in that] they may treat me…more as a threat. If I were to ever… show frustration or anger, or slightly raise my voice, I think [I] would just get a different response than if it was a five-foot nine white guy. (P6)

Participant 13 felt white fear and discomfort with Blackness was not their problem to solve stating, “You (white people) feel awkward here (with Black people present), and I can’t really help you. So, I hope you make it out, but if you don’t, tough.”

In short, to exist in medicine participants had to resist implicit and explicit organizational biases because, as P7 put it, “This is a space that is historically reserved for a certain type of person – white male.” At the same time, their resistance included fortitude to continue to exist in this space, despite all of the attempts that others made to rip the thread that sewed participants into medicine’s tapestry. Pieced together, this aspect of the quilt shows that residents’ darker tones and textures sat in contrast to the white fabric surrounding them. The stitches piecing the two colors together were loose. This block of the quilt stayed together only because of the strength and thickness of the darker fabric gripping the thread.

### Future Generations of Black Physicians and Patients: “… *professional resistance is probably going to define the rest of my career*”

Black futures were the second block of the quilt. Every participant noted they planned to resist in their future careers in at least these two ways: improving the equity in the treatment of Black patients and facilitating opportunities for Black people to enter medicine. Taking care of Black patients was cited as one of the main reasons these trainees became physicians: “One of the reasons I went into medicine is to have some sort of impact for minorities, Black people in general” (P10). The desire to improve health equity for Black patients also manifested through specialty choices; P4 explained, “Knowing that [medical condition] affects African Americans more, I felt like I should be more interested in it or I should go into that as a Fellowship.” The value of collectivism or community-focused career decision making was tightly sewn onto the quilt block with multiple repeated stitches.

Participants were future oriented, taking multiple approaches to ensuring other Black people could enter and excel within medicine. P12 felt that facilitating opportunities for Black physician trainees “…creates a sense of community for people that are trying to … enter into the space as well.” Participants served as role models for trainees new to the program. P9 recalled a situation where another trainee used hairstyle as a code for asking if the program was safe in this environment,

I remember a student came to our program and asked me if anyone has ever told me anything about my hair… I knew what the question really was: do they give you a problem about it? And I told her no. And I think just that small interaction made her feel comfortable. That she can be here, that she can express herself the way she wants to if she sees someone else being able to do that. So, I think that shows how beneficial it can be to have people of color in these positions. (P9)

Although there was a lot of sharing about facilitating and sponsoring other Black people in medicine, this strip of fabric also had loose threads. Participants were not sure if they could continuously push through the toxicity towards potentially good outcomes. P12 reflected, “I’m gonna have to be the one to do it, and so in terms of career trajectory, it just puts me in a place of ‘Do I want to keep doing this?’” They further expounded,

I pay special attention to Black residents. I interact with my [Black] patients differently, because I know that most likely in their space, they are [experiencing] similar resistance. The funny thing is, I think my ability to do that is impaired by the fact that I’m having to do my own professional resistance. So, it becomes a cycle. (P12)

Participants recognized that the experience of resistance was more stressful in academic medicine. P7 explained “You shouldn’t have to feel as if [you’re] the first one here that looks like [you],” and shared frustration about needing to teach white trainees and faculty “how to treat me.” This participant concluded their thoughts with, “I’m tired of academia. I need to get out” (P7). Although participants hoped to leave a legacy of community and mentorship for future Black trainees, moving from existing as a form of resistance to specific acts of resistance, strained participants’ energy and limited their ability to achieve personal goals.

Participants’ understanding of resistance as ongoing regardless of environment was another strip of fabric with loose threads. P5 explained the importance of having an environment with both challenges and support.

I actually think that professional resistance is probably going to define the rest of my career, because I know that I cannot be completely comfortable, because I know that if I’m not resisting something that just means that I’m sheltering myself from the world, from of all of these issues. But also, I can’t be in such a shitty situation that I’m constantly resisting and I’m gonna become brittle and break. So, I’m actively thinking about what job? What location? What role gives me the right balance of comfort so that I am safe and happy and all those things, but the right amount of opportunities for professional resistance to make me feel like I’m making the world a better place?” (P5)

In this part of the quilt, we see that Black residents viewed the entirety of their career in light of how participants will undergo the experience of professional resistance. Resistance within this block shifts away from just successfully completing training into long-term ideas of how participants will continue to push for new patterns in the tapestry of medicine.

### Racial Battle Fatigue: *“Can a brother slip… without the entire race being in jeopardy?”*

The third block of the quilt was made from strips that Smith (2007) characterized as racial battle fatigue. Specifically, Blackness was frequently called into question within the profession, and they needed to defend themselves and their communities in ways that were nearly impossible. P3 explained,

If a white person does something stupid, it’s just that white person right? It’s just that one bad egg, right? But, by the time you start moving up in the echelon of society, and you’re a Black person, and you are doing something stupid, all of a sudden it is the entire Black [population] that is doing something stupid. So, now you’re a representation and so there is more pressure for you to be uptight, right? For you to know your shit, right? Like, people are gonna question what you say. People are going to question your authority. And so you gotta come out correct every fucking time, right? And some days that’s the motivation, it’s like, you will never catch me slipping, right? …Can a brother slip… without the entire race being in jeopardy? (P3)

P7, who raised the same concern, pointing to the larger issue of resistance as a source of energy depletion because they needed to think about the next generation, their own safety in an unsafe environment, all the while learning to be a physician:

You can’t mess up because if you do they’re not gonna let another one of us in here. It … can be burdensome… I just want to just be me and not necessarily be a token representation for everybody that looks like me that may come after me in this space. (P7)

This part of the quilt showed patterns of never being able to have a day off from resistance. It is an ever-present experience, surrounding the individual. As a Black resident, not only do they have to resist everything in their environment trying to overtake and extinguish their presence, they also need to be perfect. In other words, each quilting block made by a Black quilter needs to be perfectly square; measured and cut from high quality cloth. If there are any defects, such as threading or asymmetry, the whole tapestry is in danger of never including Black artistry into the pattern. The consequence is that the tapestry will be made with the exclusion of Black techniques that will be replicated in perpetuity.

The responsibilities surrounding resistance on top of being a trainee were heavy and burdensome to many of the participants. P5 described how each decision held time and energy costs.

This is unfortunately part of the minority tax. I feel like as minorities… it’s our double responsibility to make the workplace… a better place, but also to make it more welcoming for us, for the people that we serve, and for the people who are coming in after us… it’s a huge burden that we all carry. And even if we don’t act on it, that burden is still there, because everyone else expects you to give the DEI presentations and if you say ‘no’ that has its consequences. If you say ‘yes,’ it has consequences… So it’s a strained relationship. So, in summary, I think that as a Black resident my relationship with professional resistance is having to engage in it whether I like it or not. (P5)

These scraps of fabric used to fill out this part of the quilt show there is also significant mental labor and emotional energy spent on trying to exist in a field that is already cognitively demanding, and then placed on top of this is the burden of having to think about future quilt makers and what they will include/not include in the larger tapestry. P11 explained how this burden is not shared by their white peers; “Your colleagues who don’t have to deal with these issues.” This fatigue of having to think about resisting the present and secure the future of all Black physicians is reminiscent of other experiences they have had in being Black. As several residents explained, being Black while navigating GME is an extension of how they were taught in childhood to navigate other forms of authority, such as police. They explained that around individuals who have “higher positions” they can “get physically harmed if [they] don’t act a certain way” (P9). Trying to figure out what is acceptable, while also trying to resist active harm is mentally tiring. The resident continued, sharing “Black people, in general, are still trying to figure out what’s literally safe for us to do versus what’s uncomfortable, and it’s a hard thing to really tease out” (P9). For many, this level of thinking, negotiating, and repositioning to continue to exist in medicine is exhausting, as this one explained, “Sometimes you feel crazy. Sometimes you feel like you’re losing your mind” (P1).

### Discussion

In this study, we examined Black physician trainees’ experience as they engaged in the act of resistance. Participants highlighted their existence in the clinical training space as a form of resistance in part because professionalism standards often marginalize Black trainees by imposing double standards in areas such as communication and appearance [34,35]. Consistent with existing literature, our participants described feeling constrained by these white-defined standards, which they had no other choice but to resist. For instance, Black trainees reported being subjected to heightened scrutiny regarding their communication style, whereas their white peers could communicate informally without repercussions. Similarly, appearance standards that prioritize white norms alienated them, in particular restrictions on natural hairstyles [36]. Even with efforts to conform, their experiences differ markedly from those of their white counterparts. As a result, participants faced disproportionate scrutiny, unfair treatment, underrepresentation, and biases on top of the pressure to maintain the high professional standards.

These storied experiences extend the understanding of resistance within medical education. For Black trainees, mere existence in GME spaces constitutes a form of resistance. This finding seems to represent a unique form of professional resistance seen in some HPE literature but not previously captured for GME trainee experiences [37]. Ahmed [1] describes that “resistance is not always about overt action, but often about being a body that does not “fit” within the white normative framework… To make this point very simply: whiteness becomes a social and bodily orientation given that some bodies will be more at home in a world that is orientated around whiteness” (p. 160). This is the import of P7’s statement “This is a space that is historically reserved for a certain type of person – white male.” Thus, we see how being Black existing in GME constitutes a form of resistance; it could even be thought of as the foundational “act” of resistance against Anti-Black racism. Unfortunately, most of our participants do not feel at home in their institutions. Yet, they persist in carving out space for not just for themselves but for their values as well. Despite such pressures, our study reveals that these trainees aspire to leave a legacy of inclusivity and support for future generations of Black professionals through mentorship and advocacy, as well as by working to close health equity gaps. We also observe that academic medicine offers a unique platform for fostering community and advancing equitable healthcare even though academic medicine is oriented around whiteness, not Black physician trainees.

Our research confirms that resistance against systemic racism and discrimination takes a significant toll on the emotional and mental well-being of Black physician trainees in ways that fit with the framework of ‘racial battle fatigue’ [30,32]. Racial battle fatigue includes the *experience of existing* in spaces not designed for or considerate of Black people [32]. These exhausting effects of resistance on trainees coincide with literature showing Black physicians may be overlooked for opportunities, promotions, and mentorship that are readily available to their white peers [35,38]. Our study underscores the dual burden of resilience and exhaustion that trainees endure as they navigate professional challenges while striving to maintain their authenticity and professionalism.

To improve the experience of existing in GME for Black physician trainees and reduce racial battle fatigue, medical institutions and training programs need to re-evaluate and broaden their definitions of professionalism to be more inclusive. This includes training all staff on cultural humility and responsiveness, revising policies that disproportionately affect minorities, and fostering an environment where diverse expressions of professionalism are accepted and valued. It is not enough to focus on increasing diversity. To do so without making your organization inclusive will likely lead to harm for your diverse members [39]. A recent review by Lam et al. [38] highlights that most training programs approach issues of race superficially instead of tackling the deeper issue of racism when exploring equity, diversity, and inclusion (EDI)-related problems. The issues are often framed as numerical problems, leading to solutions that focus on increasing minority representation without delving into the experiences of learners facing racism and how systemic and interpersonal power differences disproportionately impact these individuals. Why this is difficult to accomplish is likely due to the conscious and unconscious reinforcement of white norms by individuals with agency amidst publicized efforts of organizations to improve EDI-related problems. Jayakumar & Grummert [4] described it this way, “Interaction between individuals and structure is constantly taking place to either combat or reinforce whiteness [40]. Just as people of color resist oppressive structures in unconscious and conscious ways, white people combat threats to white privilege and supremacy in unconscious and conscious ways” (p. 89).

Our findings also align with Poitevien et al. [39] and Lam et al [38], indicating that merely inviting learners into these spaces without addressing the power dynamics at play, can allow existing white-dominant standards to perpetuate harm and reinforce historical racism. Our results highlight the need for a more profound structural and ideological change within medical education to ensure that inclusivity goes beyond mere representation. In light of this, Lam et al advocates for critical and intersectional approaches to equity in postgraduate medical education. This involves not only addressing explicit curriculum content but also tackling the hidden curriculum that often perpetuates cultural insensitivity, biases, and inequitable practices [38].

Lastly, through Endarkened storywork, we aim to recenter the voices of Black trainees, highlighting their experiences of resistance within medical education. We found Endarkened storywork to be productive in HPE research involving certain minoritized persons. The methodology brought together “quilt blocks” of our participants present experiences considering the struggles encountered by the past in hopes of an improved future. As a research team, we want readers to know that the words and experiences of these participants are an invaluable gift to the HPE literature. Black people have been creating beautiful patchwork quilts for centuries [27]. They have also been stitching together traumas and tribulations to bring healing, comfort, and realized dreams to the future. On the surface, these participants’ professional resistance in sharing their stories involved vulnerability and pain, but also the celebration of rich legacies and traditions of Blackness. In conclusion, showing up is resistance and is sustaining their presence in historically white spaces, reclaiming a definition of professionalism that values all races and ethnicities, and strives to leave a legacy of support for future generations of Black professionals.

## Additional Files

The additional files for this article can be found as follows:

10.5334/pme.1788.s1 Appendix A. Interview Protocol.

10.5334/pme.1788.s2 Appendix B. Black Quilting Resources.

10.5334/pme.1788.s3 Appendix C. Analysis Example.

## Disclaimer

This work was prepared by a civilian employee of the US Government as part of the individual’s official duties and therefore is in the public domain. The opinions and assertions expressed herein are those of the author(s) and do not necessarily reflect the official policy or position of the Uniformed Services University or the Department of Defense. The views expressed in this manuscript are solely those of the authors and do not necessarily reflect those of the Uniformed Services University of the Health Sciences, the Henry M. Jackson Foundation for the Advancement of Military Medicine, Inc., or the United States Department of Defense.

This study was approved by Uniformed Services University prior to Executive Orders limiting work on issues of Diversity, Equity, and Inclusion (DEI).
