# Supplementary material for: “I have to resist simply to exist”: Black Physician Trainees’ Experiences of Professional Resistance
Source: Perspect Med Educ. 2025 Apr 29;14(1):208–18. doi: 10.5334/pme.1788 (PMC12047627; doi:10.5334/pme.1788)
Supplement: Appendix A. — Interview Protocol. [file pme-14-1-1788-s1.pdf]

# Appendix A: Interview Protocol

Interview Questions:

1. Tell me a little bit about yourself. Perhaps start with your life story or your career pathway.

   Confirm Demographic Questions

1. Current level of training
2. Institution
3. Age
4. Gender
5. Race
6. Ethnicity

- Interviewer will define Professional Resistance after question 1 and give the participant the opportunity to clarify for them the definition as well.
- Link 1 think from their story/career

2. As a Black medical trainee, what does ‘professional resistance’ mean to you? How would you describe what it is to someone who hasn’t heard the definition before?

3. As a Black trainee, how would you describe your relationship to professional resistance?
    a. Probe Question: Tell me as many ways that you can think of in which someone like you, can exhibit resistance?

4. As a Black medical trainee, can you tell me about a time when resistance played out in your professional life?
    a. Probe for examples
    b. How frequent do they come up?
    c. How often and in what situation do you find yourself interfacing with the experience of resistance?

5. Describe to me what kind of things are being resisted in these instances. Probe as necessary
    a. What was being resisted and what form of power was that linked to?
    b. Describe the circumstances surrounding the instance. What happened?
    c. Who was with you?
    d. What was at stake?
        i. What was your reason for resisting?
    e. Did you feel that you resisted in this instance?
        i. If you did resist, how did you resist in that instance?
            1. What kinds of strategies did you employ?

            2. Did you feel your professional resistance achieved what you had intended?
        ii. If you did not resist, why?
    f. How did this instance make you feel? & How does your racial identity play a role in this process?

6. How would you describe encountering or having to deal with “whiteness” in terms of resistance

7. Have you witnessed others that you racially identify with engaging in professional resistance?
- For each instance listed:
    a. Describe the circumstances surrounding the instance.
    b. What was at stake?
        i. What was your perceived reason they resisted?
    c. How did they resist?
    d. What do you think of their decisions in that instance of resistance?
    e. How did that make you feel? & How does your racial identity play a role in this process?

8. Overall, how does it feel to experience resistance, whether you feel like you resisted or not? Why do you think you experience these feelings?

9. How do you feel the intersectionality of your identities interplay with resistance?

10. How does professional resistance relate to your sense of vocation or broader professional responsibilities?

11. To what extent and in what ways do you anticipate professional resistance playing a role in your own future medical practice and responsibilities?
    a. How does that make you feel knowing that resistance will be playing this role going forward?
    b. Your anticipation for resistance’s role in the medical profession as a whole?

12. Are there any other comments, thoughts, or stories you would like to share on the topic of professional resistance in medicine or medical education?
    a. To what extent and in what ways do you anticipate professional resistance will play a role in the medical profession as a whole?

Definition of Professional Resistance:

Professional resistance are the acts that individuals and groups engage in to oppose and remedy oppression, harm or injustice in the workplace. Professional resistance may differ in its level of visibility and recognition. It may be carried in subtle, indirect ways or in overt, articulated actions. These acts of opposition may be individual or collective, material or symbolic, conscious or unconscious, spontaneous

or well orchestrated, local or global, grounded in everyday professional life or other relevant spheres of professional participation. These professional actions can be targeted against both the practical and discursive.

Resistance has also been defined in other ways including “being present” or simply “existing” especially when it comes to education. Black physicians are still underrepresented in the population, and black medical students underrepresented in matriculation. The historical legacy of systemic racism in education systems brings to mind the stories of Ruby Bridges and others whose first acts of resistance were being present in education. This legacy of racism still influences who is being trained and who practices as physicians. Resistance can be thought of in these terms too.
