# Supplementary material for: “I have to resist simply to exist”: Black Physician Trainees’ Experiences of Professional Resistance
Source: Perspect Med Educ. 2025 Apr 29;14(1):208–18. doi: 10.5334/pme.1788 (PMC12047627; doi:10.5334/pme.1788)
Supplement: Appendix B. — Black Quilting Resources. [file pme-14-1-1788-s2.pdf]

**Appendix B**

*Black Quilting Resources*

| *Resource* | *Key Ideas* |
| --- | --- |
| African American Quilters of Baltimore 2017 Show accessed at https://www.youtube.com/watch?v=X-g6Yc60jEo<br><br>African American Quilters of Baltimore: Each one Teach one https://www.aaqb.org/<br><br>The Women of Color Quilters Network https://wcqn.org/<br><br>Celebrating 20 Years of Quilting: The Story of Durham's African American Quilt Circle https://www.youtube.com/watch?v=E7vS_eo32Hs<br><br>Quilter's Websites https://wcqn.org/wcqn-quilters-websites/<br><br>Cultured Expressions https://www.culturedexpressions.com<br><br>Smithsonian Archives of American Art. Oral history interview with Peggie L. Hartwell https://www.aaa.si.edu/collections/interviews/oral-history-interview-peggie-l-hartwell-11503<br><br>Peggie Hartwell https://www.peggiehartwell.com/<br><br>Faith Ringgold https://www.faithringgold.com/videos-media/<br><br>Ed Johnetta Miller https://edjohnetta.com/pedagogy/<br><br>Carolyn L. Mazloomi https://carolynlmazloomi.com/<br><br>Carole Lyles Shaw https://carole-lyles-shaw-workshops.newzenler.com/<br><br>Valerie C. White https://valeriecwhite.com/<br><br>Gee's Bend https://www.soulsgrowndeep.org/gees-bend-quiltmakers | scraps, feeling peaceful, problem solving, keeping warm, connection to mothers, grandmothers, and other people, quilts were a way to express ideas, quilting as a textile art, creativity, inexpensive, able to use old cloths, new ideas, imperfection, doing things with my hands, quilt sisters, patterns from newspapers or books, quilting out of necessity, quilting for community, quilting as art, Seminole Technique, narrative quilts, seams, movement, square, rectangle, matching points, fussy cutting, story quilts, applique, Ankara (African wax prints) or African fabrics, sense of security, every quilt tells a story- regardless if it is intentional or unintentional, less time, fewer resources and less time, risk taking, threads continue, example of invisible Black labor (museum attributed quilts to owner of plantation, not enslaved women), family quilts, choose not to attach guild to our name, quilting as a curriculum, quilting as expression of politics, freedom of expression, sparking a conversation, the purpose is not the answers, once you are clear on why you make a quilt it is up to the viewer to reach their own conclusions about it, tied quits vs. stitching, not having time, persistence, intersections, Cuesta Benberry, Gee's Bend, all doing things that had touched our lives, narratives, |
