# Supplementary material for: “I have to resist simply to exist”: Black Physician Trainees’ Experiences of Professional Resistance
Source: Perspect Med Educ. 2025 Apr 29;14(1):208–18. doi: 10.5334/pme.1788 (PMC12047627; doi:10.5334/pme.1788)
Supplement: Appendix C. — Analysis Example. [file pme-14-1-1788-s3.pdf]

Appendix C

Analysis Example

Data Excerpt	Scraps	Pieces	Threads	Memo
P14: As a [PGY] two coming from DC still a junior in The Residency program. The simple Act of being black in these spaces is my form of resistance right now. It's already evolved somewhere where I feel empowered to tell my seniors or tell my attendings where something just doesn't sound right.	Being present<br>Changing<br>Year dependent			Memo Group:<br>Transcript notes<br>Date: 12/08/2023<br>Author: KB
P14: We grew up with laughter. My dad's the funniest man that I know my mom's hilarious, so if I just come to work and it's like that I'm like why would you say that someone but I was worried about that because I didn't know if they would know how to train me because of my personality and also being black I feel like you could be a white woman and act the way I could.<br>P14: Because you're louder you're allowed to crack those jokes. You're allowed to navigate a space at your own disposal. That means people listen to you and so being a black woman and also saying these jokes and also doing these things. those are riskier jokes, but I have to do it because if I don't then maybe that's the form of resistance that is actually coming out when I exist. Is that joy and that laughter and saying things how I think	Being joyful<br>Taking my bullshit over this motherfuckers bullshit.<br>White women can<br>I exist	The burden and the necessity of resistance (as a result of existence)<br><br>This is still hard so many years later	Existing in this space	1) Doing this research is a form of resistance. It will also expose you to secondary trauma.<br><br>2) P1 does a nice job of showing how the concept of professionalism can be racially weaponized.
P13: I'm gonna be honest. It's difficult because I think it happens, it's such an embedded part of the process, that isolated instances are hard to remember. I feel like I mean, I can probably come up with something and think about it but top my head. I'm just kind of like man, I feel like every day I go in and I'm hyper aware of my size, my height, the tone of my voice, how I greet people. Like and not just in a way of I'm trying to be polite, all but in the way of if I stand in the corner and I have my arms crossed and I look more serious. Like there's and I don't know that that's always like me resisting. It's just like that. I would say is just me existing, just figuring out the space where to be safe and how to blend in, but be helpful and be like that kind of never-ending balance.	Constant<br>Hypervigilance<br>Not being seen as a threat<br>Body<br>Always there<br>Embedded		Connecting from past to present to future.	3) The idea of "not getting lost" (Line 321, Page 8) seems important. I am curious if other participants also have this awareness/response to the how do you keep your sanity question?

P12: I mean it's like just being there. Just showing up to work every day is an act of resistance.<br><br>P12: it's just being at this point in my residency career- is it's kind of surreal because I remember my first few days or weeks at the program- no way- there is no way I am going to make it to 2024. Is just like this- if I have to deal with this every day- I don't know just you don't think you can make it. And then you'll go through Black History Month and you'll run across some kind of story about, one of our ancestors and the stuff they went through, and you be like bro, there's no way I'm about to buckle.<br><br>P11: I am a huge black guy walking around the Hospital taking care of patients. And the slightest thing I do can be perceived in the wrong way. So I'm very particular about those. Like how I interact with patients all that stuff how I talk to my colleagues. So it's there<br><br>P10: Complaining was just something that I didn't see in myself about this making a big stink about these aggressions and things like I felt like I said, I'm just gonna make comments. I'm gonna be quiet. I'm gonna do my job. And then I'm gonna get out of here.<br><br>P9: Particularly in the context of institutionalized racism and upholding these standards. It made me kind of shy away. from the position of leadership and kind of just wanting, leading me to feel the only way I could be true to myself be comfortable in my own skin, act the way I want to act is to not, be in that position of leadership and not be tied to an institution.<br><br>P8: I was really shocked by this line of professional resistance can sometimes be just existing which is something that I strongly agree with and it's something that I think it's harmful like self-harming and by that, I mean, I think sometimes we allow ourselves to exist in very toxic spaces out of this mission to resist and in some ways that's part of what it means to resist but is it can turn into this	Good quotes<br>Show up<br><br>Can't let it break me<br>Connecting thread to past and future<br><br>Embodied<br>Hyper vigilant<br><br>Be quiet<br>Do job<br>Go home<br><br>Not lead<br>Be myself<br>Racism<br><br>Place-based<br>Toxic<br>Existence<br>Harmful	Resistance is unavoidable<br><br>Existing shouldn't be this hard<br><br>Can be harmful or toxic<br><br>In spaces where hyper surveillance and being the only are the standard	Relationship status: complicated at best	Memo Group: Patterns<br>Date: 1/29/2024<br>Author: KB<br><br>There seems to be a pattern that happens as folks go through the PYG sequence. First, trying to keep head above water. Then, fighting battles for others. And later on learning how to protect your peace and understanding that you are the battle. You are the space of the flight.<br><br>Memo Group: Emerging Ideas<br>Date: ?<br>Author IH<br><br>Emerging Ideas:<br>1. Existing as primary form of resistance…. -> constantly reminding people<br>2. How are people defining resistance<br>3. Strained

disproportionate harm.<br><br>P7: “Being present honestly is a form of resistance. Like in my experience. I'm the second black woman in my program period.”<br><br>P7: I think just existing in spaces where people generally don't think about somebody that looks like you being present. I think it's kind of in a way kind of form of resistance -this is a space that is historically reserved for certain type of person, white male, and I was still able to essentially kind of infiltrate that space or I'm good enough to be exist in that space.<br><br>P6: “You don't really always feel it because me, simply existing is a thing. It’s a form of resistance. And if you think about it, most of the aspects of my life, are acts of resistance. Most people in [Name of Caribbean Country] are extremely poor. Most people who grew up in the area that I grew up in [Name of State in Southeast US] most Black people aren't doing as well as me quote unquote. And so eventually unless it becomes very overt, I think it's more just like routine, we bring it up now because obviously we're an interview and everything. And unless we're in settings this, it doesn't really always hit. Especially if you're in residency and you got other things to worry about so.”<br><br>P5: “Like during didactics, cultural stuff was brought up. And immediately everyone's eyes turned at me. And it's like even if I stayed completely silent in that moment, that is a decision that I have to make. That's a burden that I'm carrying. So it's like, it's just there.. . . So in summary, I think that as a Black resident my relationship with professional resistance is having to engage in it whether I like it or not.”<br><br>P5: I think that the most powerful form of resistance though for us is really just like, and there's a whole power dynamic here, blah, blah, blah. But I feel like the biggest form of resistance for us is just being on this side of the desk [reference to being the doctor].	Only<br>Being present<br><br>Representation<br>Defy expectation<br>White spaces<br><br>Doesn’t always hit<br>Becomes overt<br>Existing is a thing<br>Most aspects of my life are resistance.<br>Other worries (learning)<br><br>No choice<br>All eyes on me<br>Decision either way<br><br>Just being here<br><br>Just being here<br>Getting through training<br><br>Being a doctor<br>Patient			relationship with resistance…<br>4. Reclaiming professionalism and reclaiming the hypocrisy of professionalism<br>5. How they are doing it? Encountering whiteness & positioning. Reclaiming professionalism Tangible ways to do resistance<br>6. Here’s how they are seeing it, how they are doing it, how they are describing and defining it (defining, experiencing, and doing)

P4: I don’t know that I have a great, visualization of what resisted looks like, other than just going through training, and being present.<br><br>P4: Just knowing that, ummm that some patients may be taken aback that you're their physician. I feel like that's a form of resistance. Some patients may not, like ahh, think that you are as qualified because of your race or skin color.<br><br>P3: I be resisting all my life<br><br>P3: Most of my resistance is done with action, with deeds, right. And so it's it’s stuff I'm gonna do anyways.<br><br>P2: Choose yourself, because nobody else is gonna choose you. So pick yourself and what's gonna allow you to heal the most people while not destroying yourself from the inside out.<br><br>P2: When I was in residency, I think I stood out as a resident, and sometimes I feel like a bad thing in the sense. I don't know if it was because people were surprised that I was good, or I don't know what it was? But I do know that another Black resident came after me and she was compared to me all the time. We are nothing alike. Our organization styles, the way we look, where we come from. I mean nothing alike, except we're Black women. Her entire intern year was comparison to me. I think white people don't have to deal with that.<br><br>P1: Simple presence is resistance.<br><br>P1: Not losing your mind, in all of this is form of resistance.	interactions<br><br>Title of paper<br>Life long<br><br>Going to do anyways<br><br>Staying alive<br>Prioritize self<br>Protect peace<br><br>Hyper surveillance<br>Comparison<br>White people don’t have to deal with this<br><br>Being Present<br><br>Staying sane			

Note: **Note: This table is present in imperfect form, the seams are not intended to match. The columns do not line up.*
